# Social Robot Interventions in Mental Health Care and Their Outcomes, Barriers, and Facilitators: Scoping Review

**DOI:** 10.2196/36094

**Published:** 2022-04-19

**Authors:** Imane Guemghar, Paula Pires de Oliveira Padilha, Amal Abdel-Baki, Didier Jutras-Aswad, Jesseca Paquette, Marie-Pascale Pomey

**Affiliations:** 1 Centre de Recherche du Centre Hospitalier de l’Université de Montréal Montreal, QC Canada; 2 Faculté de Médecine Université de Montréal Montreal, QC Canada; 3 Département de Psychiatrie et d'Addictologie Université de Montréal Montreal, QC Canada; 4 Centre d’Excellence pour le Partenariat avec les Patients et le Public Montreal, QC Canada; 5 Département de Gestion, Évaluation et Politique de Santé École de Santé Publique de l’Université de Montréal Montreal, QC Canada

**Keywords:** social robots, socially assistive robots, SARs, mental health, mental health services, dementia, autism spectrum disorder, schizophrenia, depression, scoping review

## Abstract

**Background:**

The use of social robots as innovative therapeutic tools has been increasingly explored in recent years in an effort to address the growing need for alternative intervention modalities in mental health care.

**Objective:**

The aim of this scoping review was to identify and describe social robot interventions in mental health facilities and to highlight their outcomes as well as the barriers and facilitators to their implementation.

**Methods:**

A scoping review of the literature published since 2015 was conducted using the Arksey and O’Malley’s framework. The MEDLINE, Embase, Cochrane Central Register of Controlled Trials, and PsycINFO databases were searched, and 2239 papers were retrieved. The papers included were primary empirical studies published in peer-reviewed literature. Eligible studies were set in mental health facilities and they included participants with a known mental health disorder. The methodological quality of the included papers was also assessed using the Mixed Methods Appraisal Tool.

**Results:**

A total of 30 papers met the eligibility criteria for this review. Studies involved participants with dementia, cognitive impairment, schizophrenia, depression, autism spectrum disorder, attention-deficit hyperactivity disorder, and an intellectual disability. The outcomes studied included engagement, social interaction, emotional state, agitation, behavior, and quality of life.

**Conclusions:**

The methodological weaknesses of the studies conducted this far and the lack of diversity in the conditions studied limit the generalizability of the results. However, despite the presence of certain barriers to their implementation (eg, technical problems, unsuitable environment, staff resistance), social robot interventions generally show positive effects on patients with mental health disorders. Studies of stronger methodological quality are needed to further understand the benefits and the place of social robots in mental health care.

## Introduction

Health care needs are on the rise. Faced with a shortage of staff, equipment, and funding, the quest for innovative solutions to address these needs is thriving. Among emerging solutions, the use of robots is increasingly popular. Indeed, robots are becoming more prominent in the health industry, where they are already employed as surgery, drug delivery, and diagnosis devices [1]. Lately, the use of socially assistive robots (SARs) is attracting the interest of many researchers.

SARs (or social robots) are robots meant to provide assistance through social interaction [2]. Their built-in sound, image, and motion sensors enable them to respond autonomously to a user and his environment [3-5]. SARs can be classified into 2 categories: companion and service-type robots. Although companion robots offer psychological support to the patient, service-type robots provide functional assistance to complete daily tasks [5]. It is worth noting that while this distinction may be found in the literature, many social robots can be featured in both categories. SARs, often in animal or humanoid forms, have a variety of functionalities to engage a user’s attention [6,7]. Animal-like robots are created to reproduce the physiological, psychological, cognitive, and socioemotional benefits of animal-assisted therapy without the associated inconveniences [8-11]. Real animals can cause allergies and evoke fear in some patients [12]. Pet robots require much less maintenance and are considered a safer choice for therapy in a care setting [13]. The reduced noise level, the diminished workload requirement, and the lower costs are the additional benefits [14,15]. Pet robots generally fall into the category of companion robots. Conversely, SARs embodied in a humanoid appearance show the highest levels of acceptability and usability among participants. These robots, with humanlike facial features, communication modalities, and motion patterns, seem to create a more natural interaction [16-22]. Some can converse, play music, and display images or videos. Others may even perform movements to demonstrate a set of physical exercises to an audience. Humanoid robots are usually considered to be service-type robots.

Although research is still in its early stages, SAR interventions have been carried out in a number of areas in health care. In pediatric research, studies suggest that social robots could contribute to the reduction of pain and distress in hospitalized children [23,24]. Other studies, including participants with autism spectrum disorders (ASDs), also showed that social robots could be used to teach certain behaviors and communication skills [25-27]. More often, studies with social robots are conducted with a geriatric population. With this population, it has been determined that SARs could be used to improve physical exercise and monitor health status [28,29]. In this respect, a recent randomized clinical trial found that social robots improved the adherence to medications and rehabilitation exercises in older adults with chronic obstructive pulmonary disease [30].

Currently, research with SARs is focused on their use in mental health care. In a paper published in 2015, Rabbitt et al [7] discussed social robots’ applications in mental health care. Among numerous observations, it was pointed out that the clinical application of social robots was limited to few diagnoses. Indeed, numerous studies showed beneficial effects of social robots’ intervention on the quality of life and well-being of people with dementia [5]. Other mental health conditions were not given the same degree of consideration. It was also noted that the quality and amount of evidence available lacked strength. These elements were also raised in other reviews [31,32]. To improve understanding of how social robots have been used to help people in mental health care in recent years, we conducted a scoping review to identify the outcomes, barriers, and facilitators of SAR interventions. Although there have been reviews of SAR use in other health care contexts, reviews solely focused on mental health care are lacking [33-35]. Furthermore, recent reviews on SARs have either limited the scope of their review to a precise diagnosis, to an exact type of robot, or to a population of certain age [36-39]. To fully understand how social robots could be used in mental health care, we chose to avoid such limits.

## Methods

The PRISMA-ScR (Preferred Reporting Items for Systematic Reviews and Meta-Analyses extension for Scoping Reviews) checklist was used as a guideline to ensure the methodological transparency of this review [40]. This scoping review was conducted following the Arksey and O’Malley’s framework [41]. The framework consists of the following 5 stages.

### Stage 1: Identifying the Research Questions

This scoping review addresses the following research questions:

1. What types of social robots have been used in mental health care in the past years?
2. What were the outcomes of social robot interventions?
3. What were the barriers and facilitators of their implementation?
4. Based on the results of our scoping review, what aspects require further research?

### Stage 2: Identifying Relevant Studies

#### Eligibility Criteria

The following eligibility criteria were established to guide the literature review:

1. Date of publication: The field of robotics is ever changing, and improvements are made at an astonishing speed. The limitations identified several years ago are not the same as those currently encountered. Since we wanted an up-to-date portrait of the use of social robots in mental health care, we reviewed all publications only from 2015 to the present.
2. Language of publication: The language of the studies was restricted to English.
3. Study design: Included papers were restricted to primary empirical studies (eg, quantitative, qualitative, or mixed methods) published in the peer-reviewed literature. Publications were excluded if they were considered gray literature (eg, reports, theses, newsletters).
4. Setting: Eligible studies were set in mental health facilities. Hospitals and nursing homes were included. Studies set in patients’ own homes were excluded. Studies set in schools were also excluded.
5. Population: Participants of eligible studies had a mental health disorder. A mental health disorder was defined as the existence of a clinically recognizable set of symptoms or behavior associated in most cases with distress and with interference with personal functions according to the International Classification of Diseases, 10th edition [42]. No restrictions were applied on the population age.
6. Program of care or intervention: Eligible studies implemented an evidence-based social robot program or intervention in mental health facilities. Teaching programs involving social robots were excluded. For instance, interventions using robots to teach communication skills to participants with ASDs were excluded. Brain training programs where robots provided exercises to improve cognition and memory in people with dementia were also excluded for the same reason.

#### Search Strategy

The following electronic databases were searched using the Ovid research platform: MEDLINE, Embase, Cochrane Central Register of Controlled Trials, and PsycINFO. The search strategy was developed in Ovid MEDLINE. It consisted of keywords and subject headings (Textbox 1). It was subsequently adapted for other databases. The final search strategy was validated by an experienced librarian to ensure that the literature was covered in a comprehensive manner. The electronic databases were first searched on March 26, 2021 and then searched again on November 2, 2021.

**Search strategy in Ovid MEDLINE.**

[psychology.fs. AND Robotics/] OR Self-Help Devices/px [Psychology] OR companion robot* OR social robot* OR human* robot* OR robopet* OR (Social* adj2 robot*) OR (pet* adj1 robot*) OR (therap* adj1 robot*) OR (animal* adj1 robot*) OR non-human* robot* OR (interacti* adj1 robot*)

AND

psychiatr* OR dementia OR schizophreni* OR autis* OR depress* OR isolat* OR solitude OR alzheimer* OR mental* OR psycholog* OR anxiet* OR Neurodegenerative Diseases/px [Psychology] OR exp Mental Health/OR exp Mental Disorders/OR exp Mental Health Services/OR Mental Healing/px [Psychology] OR exp Psychiatry/OR psychology/OR psychology, positive/OR psychology, adolescent/OR psychology, child/OR cognitive science/OR psychology, developmental/OR psychology, clinical/OR psychology, comparative/OR psychology, educational/OR psychology, experimental/OR psychology, medical/OR psychology, social/OR exp Autism Spectrum Disorder/OR exp Anxiety/OR exp Schizophrenia/OR exp Psychotic Disorders/OR exp Neurocognitive Disorders/OR exp Dementia/OR Hospitals, Isolation/OR Depression/OR exp Anxiety Disorders/OR Cognitive Dysfunction/

### Stage 3: Study Selection

**Figure 1:**
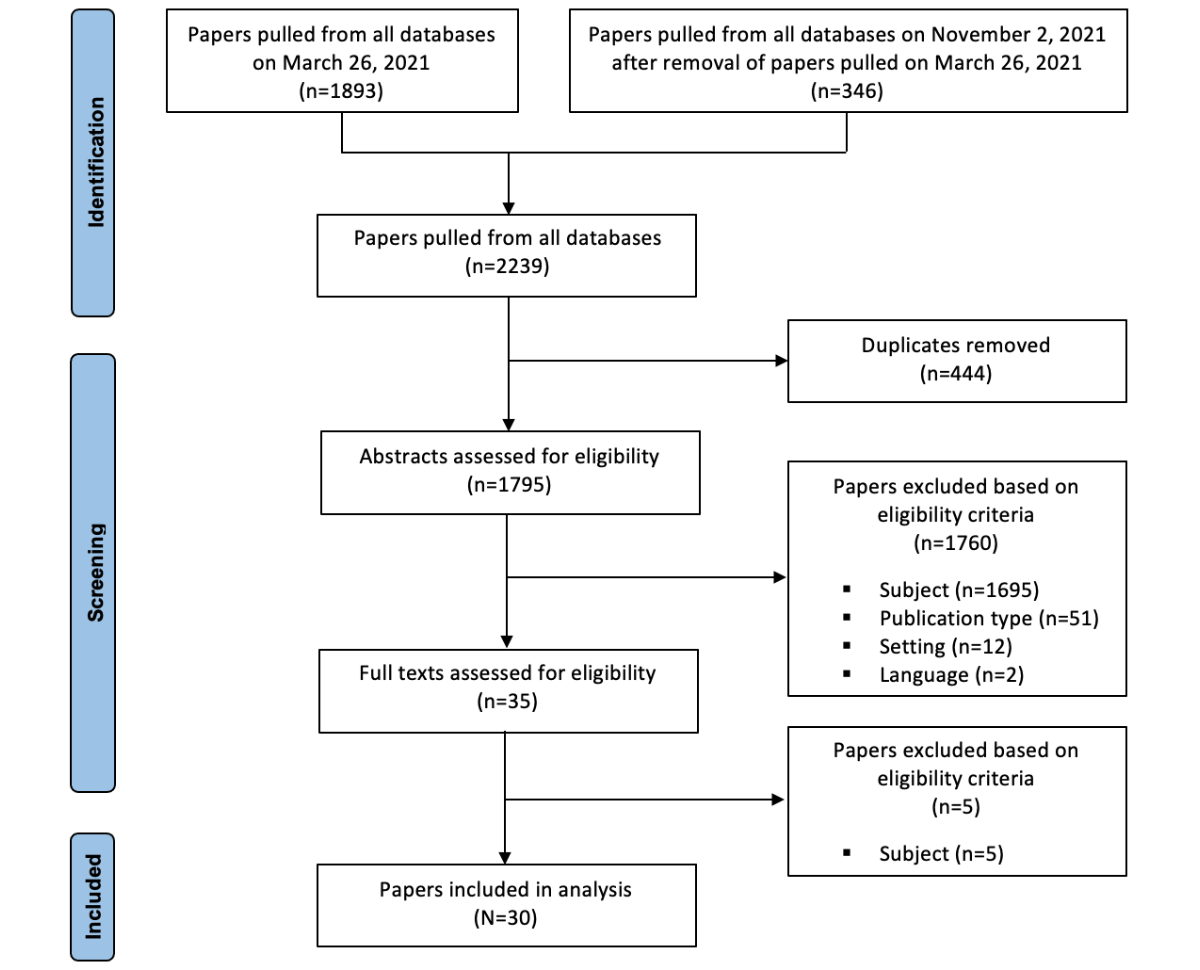
PRISMA (Preferred Reporting Items for Systematic Reviews and Meta-Analyses) flow diagram.

Screening was carried out using the Rayyan reference management tool. After duplicates were removed, 2239 titles and abstracts were assessed for eligibility by 2 independent reviewers (IG and JP). To confirm understanding of the eligibility criteria, screening of the first 50 papers was pilot tested. If necessary, the criteria were redefined to ensure consistency between the reviewers. Subsequently, the full texts were evaluated to confirm inclusion. A senior reviewer (MPP) was consulted when consensus could not be achieved through discussions, and all exclusions were documented. Thirty papers were included in the scoping review. The screening process is detailed in the PRISMA flow diagram shown in Figure 1.

### Stage 4: Charting the Data

The 30 papers selected for this review were tabulated in Microsoft Excel. A data extraction grid was developed, and 2 reviewers collected the data. The following data were extracted from the selected papers: (1) descriptive characteristics (eg, author, year, country, publication date, setting, study design, participants’ characteristics), (2) social robot interventions and outcomes, (3) implementation strategies as defined by the Effective Practice and Organization of Care (EPOC) taxonomy [43], and (4) barriers and facilitators encountered during the implementation as defined by the Consolidated Framework for Implementation Research (CFIR) [44]. The methodological quality of the included studies was assessed using the 2018 version of the Mixed Method Appraisal Tool [45]. Each publication was assessed independently by 2 raters (IG and PPdOP). Differences in appraisal were discussed until consensus was reached.

### Stage 5: Collating, Summarizing, and Reporting the Results

The characteristics of the included studies (eg, author, year, publication date, study design/method, participants’ characteristics) were described. Interventions and their outcomes were summarized and tabulated. Tables were also used to present implementation strategies as well as barriers and facilitators.

## Results

### Characteristics of the Included Studies

Thirty papers were included in this scoping review [8-10,16,18-22,46-66]. Studies used 15 different social robots. Eighteen studies used animal-shaped robots, among which the PARO seal robot was used most often (n=12), followed distantly by the AIBO dog robot (n=2). Two studies also used cat robots of different brands: JustoCat (n=1) and Joy For All (n=1). One study used both cat and dog Hasbro robots. Finally, 1 study used a robotic sheep. Seven studies used humanoid robots: NAO (n=2), CommU (n=1), Kabochan (n=1), MARIO KOMPAÏ (n=1), Pepper (n=1), and Telenoid (n=1). Three studies used other types of robots: Chapit (n=1), CuDDler (n=1), and PaPeRo (n=1). The included papers were published between 2015 and 2021 in a variety of peer-reviewed journals (5 were published in 2015, 3 in 2016, 5 in 2017, 3 in 2018, 3 in 2019, 9 in 2020, and 2 in 2021).

Ten publications were quantitative nonrandomized studies, 8 were designed as randomized controlled trials, 6 were qualitative studies, 5 were mixed method studies, and 1 was a quantitative descriptive study. Studies were set in Australia (n=5), Japan (n=5), Netherlands (n=3), United States (n=3), Norway (n=1), Taiwan (n=2), Canada (n=1), China (n=1), France (n=1), New Zealand (n=1), Spain (n=1), Sweden (n=1), and Kazakhstan (n=1). Three publications reported on a multicenter study set in Ireland, Italy, and in the United Kingdom.

The sample size of the included studies ranged from between 1 and 415 participants. In 2 studies, the participants were children. In another study, participants were adults of various ages. The other 27 publications reported on studies conducted on a geriatric population. In accordance with the eligibility criteria, all studies involved participants with a mental health disorder. Twenty-four papers reported on participants with dementia. Other studies involved participants with a cognitive impairment (n=2), schizophrenia (n=2), depression (n=1), ASD (n=2), attention-deficit hyperactivity disorder (n=1), and intellectual disability (n=1). A catalog of the included papers [8-10,16,18-22,46-66] describing studies, samples, interventions, and main findings collected in our review is available in Multimedia Appendix 1. The EPOC implementation strategies discussed in each paper are compiled in Table 1. Note that the terms “education” and “educational” do not refer to the intervention but rather to the implementation. For example, educational meetings may refer to training sessions during which the functions of the robots are explained to the staff involved in the intervention.

**Table 1:** Effective Practice and Organization of Care implementation strategies discussed in the included papers.

Implementation	References for the included papers
Communities of practice	[10,18,56]
Continuous quality improvement	[47]
Educational games	[59,62,63]
Educational materials	[9,16,49,51,55,56,62-64,66]
Educational meetings	[8,9,16,46,49,51-54,57,60,64-66]
Educational outreach visits or academic detailing	[16]
Interprofessional education	[66]
Local consensus processes	[8,9,16,22,46,48,49,52,55,57,60,61,65,66]
Managerial supervision	[51,53-56]
Patient-mediated interventions	[10,16,18,21,48,54,57,59,61,63-65]
Routine patient-reported outcome measures	[10]
Tailored interventions	[18,21,22,48,49,53,56,57,59,62,65,66]

### Mental Health Outcomes

In most cases, studies assessed the impact of social robots on engagement, social interaction, emotional state, agitation, behavior, and quality of life. The majority reported positive results on patients’ quality of life, including reduced loneliness and isolation [18,48,51,59] and improvements in mood and anxiety [9,18-20,48,53,56,60,61,66] and agitated behaviors [9,47,52]. Feelings of comfort or reduced stress following social robot interventions were also described [51,52,66], although 1 study including participants with cognitive decline showed changes in the electroencephalogram, which were indicative of increased stress [50]. In some studies that focused on participants with dementia, SARs appeared to increase social engagement between patients, caregivers, and family members [8,16,18,20,21,47,49,52,53,58]. Further, SARs were emphasized as an alternative to alleviate the burden of caregivers, since they could free up time allowing carers to partake in other professional or daily tasks [16,20,47,63]. Furthermore, the use of social robots could enhance communication skills and improvements in joint attention among children with ASD, as described in the study by Kumazaki et al [22].

### Barriers and Facilitators to the Implementation of Social Robots in Mental Health Facilities

Some barriers and facilitators were identified in the 30 included publications, using the CFIR as a guide to present the results in an adapted form. Three of the 5 domains in the CFIR were identified in this study: intervention characteristics, which refers to the key attributes of the intervention, called by the authors as “technical category;” inner setting, which refers to the features of the implementing organization, called by the authors as “organizational category;” and the characteristics of the individuals involved in the implementation, called by the authors as “clinical category.” A summary of these is presented in Table 2.

**Table 2:** Barriers and facilitators to the implementation discussed in the included papers.

Factors				References of the papers
**Barriers**				
	**Organizational**			
		Noisy environment during interaction	[16,18,48]	
		Storage area necessary	[46]	
		Charging necessary	[46]	
		Hygiene measures necessary	[46]	
		Staff/caregivers resistant to implementation	[18,66]	
		Increased workload for staff/caregivers	[8,66]	
		Frequency of sessions not adapted to patients’ needs	[61]	
	**Clinical**			
		Participants with an advanced cognitive decline	[18-20,54,66]	
		Participants with a hearing impairment	[20,63]	
		Difficult disengagement after the robot’s removal	[55]	
		Risk of deception	[49,51,66]	
		Participants with a language impairment	[50]	
		Interaction with the robot seemed infantilizing	[8,51]	
		Participants feared the robot	[22]	
		Participants misunderstood the purposes of the study	[8]	
		Frustrating interruption of activities	[57]	
	**Technical**			
		Robot was difficult to understand	[16,21,63]	
		Robot’s touchscreen was difficult to use	[16,48]	
		Robot’s voice recognition system was deficient	[18,48]	
		Limited visibility of the robot’s screen display	[21]	
		Robot’s speech rhythm deficient (too fast, long pauses, etc)	[21,62,63]	
		Robot was too noisy	[8,56,61]	
		Connection between devices was unstable	[8]	
		Robot was fragile	[8]	
		Robot was heavy	[8,61]	
		Robot was too big	[8]	
		Robot interrupted conversations	[63]	
		Robot spoke a limited number of languages	[19,48]	
**Facilitators**				
	**Organizational**			
		Staff/caregivers had a positive perception of the robot	[18,46,66]	
		Staff/caregivers received training	[8,9,16,22,46,49,51-55,57,60,64-66]	
		Staff/caregivers promoted the use of the robot	[46,66]	
		Robot was easily available	[10,47]	
		Low cost	[10,19,47]	
		Robot was named by participants	[55]	
		Demonstration at the beginning of the intervention	[21,53,56]	
		Intervention did not replace usual activities	[49]	
		Hygiene measures were easily applicable	[51]	
		Participants were given ownership of their robot	[10]	
		Cleaning protocol was developed	[66]	
		Sessions were carried out in a quiet separate room	[8,53,54,56]	
		Exclusion of patients uninterested by the robot	[53,54]	
		Activities with the robot were organized (eg, bingo, listening to music)	[53]	
		Verbal/written instructions for staff/caregivers	[53,56]	
		Length of sessions were flexible	[16,56]	
		Facilitator was present during sessions	[8,49,56,62,63]	
	**Technical**			
		Robot’s appearance was pleasing	[10,16,18,21,22,47,51,61]	
		Addition of stylus pen to facilitate the use of the robot’s touchscreen	[16,48]	
		Robot was easy to use, little training required	[21,47]	
		Robot was responsive to patients’ touch	[9,47,66]	
		Robot’s speech modalities were adequate	[61,66]	
		Robot was voice- and face-activated	[21]	
		Robot’s sound was clear	[10,21,22,62]	
		Robot’s voice/face recognition feature was adequate	[21]	
		Contextual interaction (intervention within augmented reality display)	[49]	
		Robot had entertaining features (apps, images, music)	[16,18,21,48,63]	

Barriers to implementation were primarily related to the characteristics of social robots, such as their physical attributes (eg, weight, size, sound, overall appearance) [8,56,61]. Technical issues (eg, connection instability, fragility and susceptibility to damages, deficient speech recognition, complexity of operating the touchscreen and preprogrammed functions, limited visibility of the robot’s screen display) were mentioned as barriers [8,16,18,21,48]. Furthermore, organizational, and institutional barriers in mental health facilities such as space allocation (eg, lack of an adequate space for interactions with SARs, lack of storage area), background noises during participants’ interaction, and uncertainty on how to delineate hygiene concerns were reported [16,18,46,48]. Negative attitudes toward social robots by staff and caregivers (eg, fear of job replacements by robots) were emphasized in 2 studies in our review [18,66]. However, some stakeholders developed positive perceptions toward social robots after witnessing their positive impacts, as reported by Bemelmans et al [67].

Most of the identified facilitators correspond with the identified barriers. For instance, the characteristics of the social robots, such as the robot’s appearance, ease of use, and technical functions (eg, the robot’s adequate speech modalities, the robot’s responsiveness to patients touch, the robot’s clear sound, the robot’s appropriate voice and face recognition) were seen as enablers [9,10,16,18,21,22,47,51,61,66]. Less noisy robots were less likely to distress the interlocutor, notably in children with ASD [22,62]. Further, the ability to adapt the robot’s functions to participants’ preferences and customize the modes of robot interaction through apps were identified as implementation facilitators [16,18,21,48,49,59,63]. An introduction phase with training and familiarization also facilitated greater acceptance to social robots [16,21,22,53,56,65]. Organizational and institutional facilitators such as easily applicable hygiene measures, flexibility in the number and duration of sessions to match users’ needs, and appropriate and quiet spaces for interactions were also identified as facilitators [8,16,51,53,54,56].

## Discussion

### Principal Findings

Overall, our review aimed to evaluate how social robots have been used to influence clinical outcomes in mental health care and the main barriers and facilitators encountered during their implementation. Our review includes 15 different social robots, and interventions ranged generally from positive to mixed results, although the statistical significance was not considered in some of the studies [8,10,20,22,51,59,62,64]. Most of the studies had very small sample sizes, a very brief duration, and had no follow-up measurements, which might make it difficult to conclude about the efficacy of the interventions [9,10,16,18,46-51,57,61,66]. In 2 of them, the intervention was not clearly described [47,63]. These methodological limitations were also highlighted in previous reviews of SAR use in mental health services [3,36].

Almost all of the studies included in this review focused on providing comfort, well-being, and companionship to the study participants. Only a minority used SARs to implement a specific intervention to improve patients’ self-management abilities or to address psychoeducation strategies. It must be considered that our scoping review excluded papers involving social robots in teaching-learning scenarios; thus, relevant studies might be missed. Further, most papers in our review (24 of 30) reported on interventions with participants with dementia. With this population, the main priorities in using SARs were the reduction of neuropsychiatric symptoms as well as the feeling of isolation and loneliness [9,51]. Therefore, the possibility to address companionship and improvement in daily support might be seen as a more relevant therapeutic benefit than self-managing treatment, as previously described in the literature [36,68].

One study in our scoping review raised the issue of the possible use of social robots to reduce loneliness during the COVID-19 pandemic [18]. Three different roles of SARs (ie, social utility, social identity, and social connectivity) allow social robots to create a supportive relationship capable of mitigating feelings of loneliness during quarantine and lockdown contexts [69]. Their role in promoting well-being was also highlighted as a promising avenue for those who are more vulnerable during the pandemic, particularly older adults and children [70]. Moreover, it must be considered that the demographics and the clinical characteristics of the participants influenced their needs. As most of the selected papers included older people with dementia, some particularities of this population must be raised. Some studies reported on participants with an advanced cognitive decline or with language and hearing impairments, which made it difficult to interact with the robot [18-20,50,54,63,66]. In addition, the complexity of operating the touchscreen and preprogrammed functions were also highlighted in this population [16,48]. Thus, tailoring an intervention to patients’ needs by using a personalized approach is identified as an important enabler that was also previously highlighted [36].

Assessing staff, family, stakeholders, and caregivers’ perspectives about SAR use in mental health services is another relevant aspect that should be considered. Consistent with our review, negative reactions were primarily described in some studies [71,72], but other studies also recorded how some stakeholders developed positive perceptions toward social robots after witnessing their positive impacts on patients [67,73-76]. Positive attitudes of care professionals toward SARs were reported as key facilitators to acceptability among users [73]. All these findings are consistent with those reported in a recent scoping review by Koh and colleagues [77]. SARs might potentially integrate traditional mental health care apps in an interactive social companion, providing a more engaging and dynamic platform for users [3]. In our review, some studies reported that the presence of different applications adapted and personalized to participants facilitated and sustained their engagement with the robot as well as their interactive behaviors [16,18,48,61]. Combining these capabilities with active user interaction allows SARs to deliver different interventions (eg, psychoeducation, techniques of cognitive-behavioral therapy), which can help users take greater ownership of their own health and well-being [3,78,79].

Although SARs have emerged as a promising approach across the field of mental health, they should be treated as an additional and complementary resource in mental health services and thus, poor substitutes for human contact [8,18,52,80]. Ethical concerns such as reduced human contact, emotional deception, and issues surrounding data security, confidentiality, and information privacy must be considered during the implementation of SARs in mental health services [80]. Most of the robots cannot assess a patient’s emotional state with great accuracy, and the absence of a human professional can have a negative impact on a patient’s adherence to a program [18,63,80]. Ideally, social robots should remain under the supervision of trained mental health professionals and should be used as a means of providing comfort, quality of life, and purposeful engagements [52,80].

### Strengths and Limitations

There are some strengths used in sustaining this work. First, the methodological framework was transparent and rigorous, and we searched multiple databases. Second, we consulted experts in the field of social robots as well as mental health researchers and professionals to emphasize the main points in each area. Finally, the Mixed Methods Appraisal Tool was employed to evaluate the quality of studies included in this review and a scientific and valuable implementation tool, CFIR, was used to guide the presentation of results. Nevertheless, this review has several limitations. Papers that were not published in English were excluded in this review and, as a result, relevant studies might be missed. In addition, the review aggregated only studies set in mental health facilities, and studies set in patients’ homes were excluded. This fact seemed to form a bias regarding the severity of mental health disorders that were included in this review. Although we did not limit our search to specifically a mental health diagnosis and did not define a specific age range, the bulk of our sample consisted of interventions with older adults with dementia. Therefore, the generalizability of our findings is limited by study characteristics. Moreover, most of the studies had small sample sizes, with brief and sometimes unclear interventions and poor and heterogeneous methodology, which might make it difficult to preclude conclusions about the efficacy of the interventions.

### Future Research and Practical Implications

Overall, our review has shown that the potential of social robots in mental health care is broad. However, there are still many gaps in this field. Since previous works on SAR interventions have mainly focused on older adults (ie, for the treatment of dementia) and children (ie, for the treatment of ASD), expanding the diagnosis would be a relevant option for the next steps in the research. As an illustration, attention is warranted for major depressive disorder, which has the highest lifetime prevalence among psychiatric disorders and is associated with high costs for the society [81]. In our scoping review, only 1 study had addressed the use of SARs for patients with major depressive disorder, and it found a statistically significant reduction in depression and loneliness and improvement in the quality of life [55]. However, both the small sample size and the relatively short duration of the intervention limit the generalizability of their results. Further research studies with larger samples, assessing long-term follow-up and with clear intervention protocols are needed in this field.

Furthermore, the use of SARs in different settings should be raised, notably for individuals with mental health needs living in remote areas and for those who feel stigmatized in traditional mental health care settings. In the context of rural communities and other resource-scarce areas, SARs would allow patients to receive health care remotely, thereby enabling such patients to avoid potential barriers to care such as travel or scheduling and thus improving patient outcomes. Further, the recent COVID-19 pandemic has highlighted telehealth’s potential in almost all health care settings. The possible use of social robots to reduce social isolation during the pandemic is a significant issue that could be explored [69,70]. Other psychotherapeutic strategies (eg, self-care tactics) combined with SARs should be raised in future research, which could be helpful in facilitating engagement with self-help treatment programs and users’ autonomy. Rather than developing a novel program treatment, SARs could be integrated into existing psychosocial approaches to improve the effectiveness of the intervention, such as an adjuvant in cognitive behavioral therapy. In this context, SARs could improve in real-time the monitoring and feedback for users through dynamic applications [7].

Tailored interventions aiming to fulfill the specific needs of a well-defined population should be explored, and further qualitative research (entirely user-centered) should investigate what people expect from the social robots’ roles played in mental health care. Single-case experimental designs might be a very useful starting point to ensure that different needs are met (ie, clinical, users’, engineers’, and roboticists’ goals). Improvements in methodology and study design, beyond pilot studies, and the use of psychometrically validated measures should also be taken into consideration.

Research that evaluates the implementation of SARs in mental health programs and that identifies their barriers and facilitators is also relevant when it comes to guiding the successful implementation of social robots in a real-world setting, particularly in an organizational context (eg, policy and government regulations for project planning and evaluation, the expense of robotics and the cost-versus-benefit relationship within services). The cost of mental disorders is already placing a high financial burden on individuals with mental health problems, their families, and the society in general, and creating cost-efficient robots seems to be a good opportunity in greatly reducing the cost in mental health care [82-84]. Further research in these areas, using an implementation framework, is needed. In all these aspects, it is essential for mental health professionals to work closely with patients and with robotics experts (ie, computer scientists, programmers, and engineers) to provide critical feedback on what tasks robots can reasonably do and which ones should be considered in the design of future interventions.

As the demand for mental health services increases, it is becoming imperative to find solutions to meet the growing needs. The use of social robots is a viable solution. Despite some technical flaws, advances in robotics now make it possible to offer a quality service for users. Our scoping review has highlighted the therapeutic effects of social robots in a variety of contexts. However, the methodological weakness of the studies often limits the generalizability of their results. Further studies should go beyond the framework of the pilot study in order to target the use of social robots for a well-defined case and to further potentiate the attributes of these technologies.

## Appendix

Multimedia Appendix 1 Catalog of the included studies and extracted data, sorted alphabetically by study author.

## Abbreviations

ASD: autism spectrum disorder
CFIR: Consolidated Framework for Implementation Research
EPOC: Effective Practice and Organization of Care
PRISMA-ScR: Preferred Reporting Items for Systematic Reviews and Meta-Analyses extension for Scoping Reviews
SAR: socially assistive robot
