# Supplementary material for: Social Robot Interventions in Mental Health Care and Their Outcomes, Barriers, and Facilitators: Scoping Review
Source: JMIR Ment Health. 2022 Apr 19;9(4):e36094. doi: 10.2196/36094 (PMC9066335; doi:10.2196/36094)
Supplement: Multimedia Appendix 1 [file mental_v9i4e36094_app1.docx]

**Catalog of the Included Studies and Extracted data, Sorted Alphabetically by Study Author**

| Reference | Study design | Population | Condition | Setting and Study period | Robot | Intervention | Main outcomes | Scales/ scores | Data collection | Main findings† | Mixed Methods Appraisal Tool (MMAT)†† |
| --- | --- | --- | --- | --- | --- | --- | --- | --- | --- | --- | --- |
| Barrett et al, 2019* [16] | Quantitative descriptive study | N: 10 (7 women/ 3 men)  Age (years): 83 (mean) | Dementia | Nursing home  Study period unclear | MARIO KOMPAÏ (humanoid robot) | 3 sessions (60 min)/week for 4 weeks. Sessions were stopped earlier if necessary. | Outcomes: Depression, social support, quality of life, usability of MARIO | Cornell Scale for Depression in Dementia (CSDD)  Multidimensional Scale of Perceived Social Support (MSPSS)  Quality of Life–Alzheimer’s Disease (QoL-AD) Scale | A staff nurse completed the CSDD at baseline and at 4-week follow-up for each participant. Participants completed the MSPSS and QoL-AD scale at baseline and at the 4-week follow-up. To assess usability of robot, participants completed a Bespoke Questionnaire and researchers completed an Observations Questionnaire after each session. | No statistically significant change in depressive state (*P=*.80), social support (*P=*.78) and quality of life (*P=*.61). It was observed that MARIO increased enjoyment and social interactions with others. | Quantitative descriptive study 4.1. Yes (sampling strategy is explicit and relevant) 4.2. Yes (sample is representative of the target population) 4.3. Yes (measurements are appropriate to answer the research question) 4.4. Yes (low risk of nonresponse bias; reasons for nonresponse are reported) 4.5. Yes (statistical analysis is appropriate to answer the research question) |
| Bemelmans et al, 2015 [46] | Quantitative non  randomized study | N: 71 (57 women/ 14 men)  Age (years): Unclear | Dementia | Small-scale care units spread over 6 locations, in 3 Dutch care institutions for intramural psychogeriatric care  May 2012 to October 2013 | PARO (seal robot) | Study divided into 4 phases (1 month each). Phase 1 and 3: participants received usual care. Phase 2 and 4: 5 sessions with the robot (15 min).  Two possible interventions: therapeutic or care support intervention. Care support intervention were attributed to patient with a specific problematic behavior (observed by care providers). | Outcomes: Short term therapeutic effect, facilitation of the daily care delivery by staff | Individually Prioritized Problems Assessment (IPPA) score  5-point mood scale (to assess psychological and psychosocial functions) | IPPA score and mood scale were completed by care providers 5 times /month. | Therapeutic interventions showed significant effects (*P<*.001). Care-support interventions showed no significant effect (*P=*.58). | Quantitative nonrandomized study 3.1. Yes (participants are representative of the target population) 3.2. Yes (measurements are appropriate to answer the research question) 3.3. No (outcome data are not complete; 71 of 91 participants completed the study) 3.4. Yes (confounders are accounted for; differences between subgroups were measured) 3.5. Yes (intervention was administered as intended) |
| Brecher, 2020 [47] | Qualitative study | N: 1 (1 man)  Age (years): 90 | Dementia (with terminal restlessness) | Veterans Affairs Community Living Center  Study period unclear | Joy For All (cat robot) | Unclear; use of robot for about a week while the patient experienced terminal restlessness | Outcome: Distress due to terminal restlessness | No scales/scores used to measure outcome | Observation by care providers | The use of robotic cat had a minimally positive effect on the patient's agitation and physical aggression. | Qualitative study - Case study 1.1. Yes (the qualitative approach is appropriate to answer the research question) 1.2. Yes (data collection method is adequate to address the research question) 1.3. No (no data analysis method is employed) 1.4 Can't tell (it is unclear if the interpretation of results is sufficiently substantiated by data) 1.5 Can't tell (the links between data sources, collection, analysis and interpretation are unclear) |
| Casey et al, 2020* [18] | Qualitative study | N: 38 (24 women/ 14 men)  Age (years): 55-93 | Dementia | Hospital (Italy), Long-term care (Ireland), Central testing center (UK)   Study period unclear | MARIO KOMPAÏ (humanoid robot) | Unclear, varied according to site. Session averaged 43,7 min in the hospital (Italy), 35 min in long-term care (Ireland) and 60 min in the community (UK). | Outcome: Perceptions and experiences of people with dementia and key stakeholders regarding the use and impact of MARIO | No scales/scores used to measure outcome | Semi-structured one-to-one interviews. | Participants answered positively to the robot. Robot's user showed an increase in engagement, attention, autonomy, and social interactions as well as a decrease in loneliness and isolation. Some participants stated MARIO improved their mood. Carers eventually saw value in the use of MARIO, although the hospital staff expected more autonomy from the robot. | Qualitative study - Qualitative description 1.1. Yes (the qualitative approach is appropriate to answer the research question) 1.2. Yes (data collection method is adequate to address the research question) 1.3. Yes (findings are adequately derived from the data; a coding framework was used) 1.4. Yes (interpretation is sufficiently substantiated by data) 1.5. Yes (links between qualitative data sources, collection, analysis, and interpretation are coherent) |
| Chu et al, 2017 [21] | Quantitative non  randomized study | N: 139 (44 women/ 95 men)  Age (years): 65-90 | Dementia | 4 Long-term care facilities   2010 - 2014 | PaPeRo (non-humanoid robot) | Participants attended 1 trial (4-6 hours) each. 28 participants attended two trials. | Outcomes: Behavioral reactions of people with dementia and selected staff | Modified Dementia Care Mapping (DCM)  Modified Well-Being/Ill-Being Scale (WIB) | Each 5 minutes, researchers assessed whether participants were engaged or not. Each 5 minutes, Behavioral reaction was assessed and assigned a code: AR - Approaching robot, IR - Interacting with robot, IO - Interacting with others, P - Pleasure.   Staff were evaluated with a 2-point scale (Less/more involvement in participants’ activities, Less/more attention to monitoring participants' reactions | Participants showed a positive engagement when interacting with the social robot, leading to an improvement in the participants' social capabilities. For instance, social engagement improved significantly between 2013 and 2014 (*P<*.05). Participants were very interested in engaging in group activities. Improvements made through the years could explain why participants approached the robot and expressed more pleasure from their interaction with it. Interaction with the robot and with others increased through the years (*P<*.05).   Caregivers were more involved and more attentive to patients' needs during activities. | Quantitative nonrandomized study 3.1. Yes (participants are representative of the target population) 3.2. Yes (measurements are appropriate to answer the research question) 3.3. Yes (outcome data are complete, all participants completed the study) 3.4. Yes (confounders are accounted for) 3.5. Yes (intervention was administered as intended) |
| D’Onofrio et al, 2019* [48] | Quantitative non  randomized study | N: 38 (24 women/ 14 men)  Age (years): 55-93 | Dementia | Hospital (Italy), Long-term care (Ireland), Central testing center (UK)   Study period unclear | MARIO KOMPAÏ (humanoid robot) | Intended intervention is unclear. Frequency and duration of interactions with MARIO varied. Participants participated to 1 to 12 sessions. Session averaged 43,7 min in the hospital (Italy), 35 min in long-term care (Ireland) and 60 min in the community (UK). | Outcomes: Engagement, acceptability and efficacy of the MARIO companion robot on clinical, cognitive, neuro-psychiatric, affective and social aspects and on resilience capacity and quality of life in patients with dementia. Burden on caregiver was also assessed. | Observational Measurement of Engagement (OME)  Cornell Scale for Depression in Dementia (CSDD)  Multidimensional Scale of Perceived Social Support (MSPSS)  14-item Resilience Scale (RS-14)  Quality of Life in Alzheimer’s Disease (QOL-AD),   Caregiver Burden Inventory (CBI) | OME was assessed during the engagement with MARIO (observation period between 3 and 15 min). Other scales/scores were administered before and after interactions with MARIO. | Engagement with MARIO varied greatly across all 3 pilot sites. Hospitalized patients showed a lower level of enthusiasm and attentiveness toward MARIO. Patients in residential care were more expressive. Patients from residential care and hospital care were more likely to interact with staff during the intervention with the robot.   Overall, the difference in CSDD (*P=*.10), QoL-AD (*P=*.08), MSPSS scores was not significant (*P=*.44). There was, however, a significant improvement in resilience score (RS-14) (*P=*.02).  55-67 years old: Significantly less depressed (CSDD) (*P=*.03) and more resilient (RS-14) (*P=*.003)  68-76 years old: Significantly better total social support perception (MSPSS) (*P=*.02) and friend support perception (MSPSS Fri) (*P=*.01).  77-85 year old : Significantly better family support perception (MSPSS Fam) (*P=*.02) | Quantitative nonrandomized study 3.1. Yes (participants are representative of the target population) 3.2. Yes (measurements are appropriate to answer the research question) 3.3. No (outcome data are not complete; 30 of 38 participants completed the study) 3.4. Yes (confounding factors are accounted for) 3.5. No (intervention was not always administered as intended) |
| Demange et al, 2018 [9] | Quantitative non  randomized study | N: 17 (14 women/ 3 men)  Age (years): 77-95 | Dementia with different behavioral and psychological symptoms of dementia (BPSD) | Geriatric hospital   January to June 2016 | PARO (seal robot) | 2 sessions (15 min)/week for 2 weeks | Primary outcomes: Emotional well-being  Secondary outcomes: Robot's usability and perception | International Positive and Negative Affect Schedule Short-Form (I-PANAS SF)  System usability scale (SUS) | Observations and scales/scores were administered Pre and Post interactions with Paro by a trained neuropsychologist.  I-PANAS SF Score were assessed twice: on day 2 (15 min) and day 12 (20 min).   Usability was assessed after the intervention with the System Usability Scale (SUS) | Significant improvement on positive affect scores (*P=*.02). No significant decrease in negative affectivity (*P=*.19).  Acceptance was generally positive amongst participants. Usability positively affected the impact of the intervention: patients communicated with the robot and displayed affective behaviors. | Quantitative nonrandomized study 3.1. Yes (participants are representative of the target population) 3.2. Yes (measurements are appropriate to answer the research question) 3.3. Yes (outcome data are complete; 15 of 17 participants completed the study) 3.4. Yes (confounders are accounted for) 3.5. Yes (intervention was administered as intended) |
| Feng et al, 2020 [49] | Quantitative randomized controlled trial | N: 16 (12 women/ 4 men)  Age (years): 85.3 (mean) | Dementia | Residential home  Study period unclear | LiveNature (ambient display with sheep robot) | 1 session (up to 20 min)/week for 4-week  Condition 1: Interaction with a proactive robot in a dynamic context;  Condition 2: participants interacted with a static robot within a dynamic context;  Condition 3 (control): Participants in a dynamic context without a robot in hand.   Participants experimented the effects of the conditions 1 or 2. All participants experimented condition 3. | Outcomes: Engagement, affective states, apathetic behavior | Observational Measurement of Engagement (OME)  Engagement of a Person with Dementia Scale (EPWDS)  Observed Emotional Rating Scale (OERS)  People Environment Apathy Rating Scales–Apathy subscale (PEAR–Apathy subscale) | OME and OERS were rated by an observer after each session. EPWDS and PEAR-Apathy subscales were completed by a trained research assistant. | Participants taking part in the first condition had a better attitude when interacting than those taking part in second condition (*P=*.049). The interaction's duration was the lowest during for those in second condition (static robot). Participants using the active robot were significantly more engaged in visual (*P=* .005), behavioral (*P<* .001), and social (*P=* .002) aspects of engagement and engagement in general (*P=* .006). Affective states (OERS) did not improve significantly in situations with the robot (static or proactive). Condition using the proactive robot generally showed a decrease in apathy-related behavior compared with control. | Quantitative randomized controlled trial  2.1. Can't tell (the way randomization was performed is unclear) 2.2. Yes (groups were comparable at baseline) 2.3. No (outcome data are not complete; 16 of 21 participants completed the study) 2.4. Yes (outcome assessors were blinded to the objectives of the study) 2.5. Yes (participants adhered to the assigned intervention) |
| Goda et al, 2020 [50] | Quantitative non  randomized study | N: 28 (17 women/ 11 men)  Age (years): 79 (mean) | Cognitive decline | 2 Day-service centers  Study period unclear | Chapit robot (teddy bear robot) | 1 session (5 min) | Outcomes: Immediate effects of robot-assisted activity on psychologi-cal and neurophysio-logical indices | 5-point Likert scale developed to evaluate emotion and mood. | The Likert scale was completed after the session. Resting-state EEG and salivary cortisol levels were assessed both before and after each session. | Participants with cognitive decline were more uneasy after the session with Chapit compared with participants with no cognitive decline. Participants in the control group felt significantly more enjoyment than the cognitive decline group (*P<*.01). EEGs of the cognitive decline group suggested an increase of stress after session. For instance, relative power index (alpha/beta) on Pz decreased significantly in cognitive decline group after the robot-assisted activity (*P<*.05). Changes in salivary cortisol levels were significantly higher in the cognitive decline group (*P=*.04). | Quantitative nonrandomized study 3.1. Yes (participants are representative of the target population) 3.2. No (measurements are not appropriate to answer the research question; scale was not tested for its validity, control of artefacts on EEG could not be achieved and cortisol levels were not measured at the optimal time) 3.3. Yes (outcome data are complete; measures were only required once) 3.4. Yes (confounding factors are accounted for) 3.5. Yes (intervention was administered as intended) |
| Gustafsson et al, 2015 [51] | Mixed method study | N: 4 (2 women/ 2 men)  Age (years): 82-90 | Dementia (late stage) | Residential mental healthcare institution   Study period unclear | JustoCat (cat robot) | Participants were able to use the robot whenever they liked for 7 weeks. | Primary outcomes: Changes in behavioral and psycho-logical symptoms of dementia (BPSD) and in quality of life.   Secondary outcomes: Usability of the robot, interaction/ impact of the robot. | Quality of Life in Late-Stage Dementia scale (QUALID)  Cohen–Mansfield Agitation Inventory instrument (CMAI). | Participants were interviewed and observed. Scale/score were administered by professional caregivers.  QUALID and CMAI were assessed 9 times. | Primary outcomes: Decrease in agitated behavior and better quality of life.    Secondary outcomes: Increase in interaction and engagement amongst participants. The robot was reliable and was used as a replacement for sedative medications.  (p-values unavailable) | Mixed method study 5.1. Yes (rationale for using a mixed methods design is adequate) 5.2. Yes (the different components of the study are effectively integrated to answer the research question) 5.3. Yes (the outputs are adequately interpreted) 5.4. Can't tell (qualitative and quantitative results were not compared since different outcomes were assessed) 5.5. Yes (the different components of the study adhere to the quality criteria of each tradition of the methods involved) |
| Hammarlund et al. 2021 [10] | Quantitative non  randomized study | N: 5 (4 women/ 1 men)  Age (years): 82.8 (mean) | Dementia | All-inclusive care center for older adults  Study period unclear | Hasbro robot (dog or cat robot) | Participants were free to use the robot whenever they liked over the course of 4 weeks. No structured sessions were elaborated. | Outcomes: Agitated behavior, quality of life, depression symptoms | Cohen-Mansfield Agitation Inventory (CMAI)  Quality of Life Assessment in Dementia (DEMQOL)  Geriatric Depression Scale Short Form (GDS-SF) | The CMAI was completed by a professional caregiver. DEMQOL and GDS-SF were completed by participants. Data was collected three times. Time 2 and time 3 marked the beginning and the end of the intervention period. | No improvement or deterioration in agitated state observed. Results indicated improvement in quality of life. Participants showed a decrease in depression symptoms across the intervention period.  (p-values unavailable) | Quantitative nonrandomized study 3.1. No (participants might not be representative of the target population, sampling strategy is not justified) 3.2. Yes (measurements are appropriate to answer the research question) 3.3. No (outcome data are not complete; data from 5 of 7 participants were analyzed) 3.4. No (confounders are not well accounted for; information about participants’ medication could not be obtained) 3.5. No (intervention was not administered as intended; compliance was inconsistent) |
| Hung et al, 2019 [66] | Qualitative study | N: 10 (4 women/ 6 men)  Age (years): 60-85 | Dementia | Geriatric mental health unit of a large urban hospital  Study period unclear | PARO (seal robot) | 2-4 sessions (20-30 min each)  Sessions with the robot were administered when the staff deemed a patient could need it. | Outcome: Overall response of hospitalized patients to robot | No scales/scores used to measure outcome | Participants were interviewed by researchers while they were using the robot. Non-verbal actions were noted.   Staff members participated to interviews and focus groups. | Data analysis showed that patients felt more social and emotional support when using PARO. Patients seemed more comfortable and relaxed. Using PARO also increased communication and social interaction. PARO was a conversation starter and served as a confidant for patients. Several participants claimed the robot made them happy. | Qualitative study 1.1. Yes (the qualitative approach is appropriate to answer the research question) 1.2. Yes (data collection methods are adequate to address the research question) 1.3. Yes (findings are adequately derived from the data, qualitative data was coded) 1.4. Yes (interpretation is sufficiently substantiated by data) 1.5. Yes (links between qualitative data sources, collection, analysis and interpretation are coherent) |
| Jones et al, 2018 [52] | Quantitative non  randomized study | N: 138 (101 women/ 37 men)  Age (years): 84 (mean) | Dementia (Alzheimer's disease, vascular dementia, dementia with Lewy bodies, Fronto temporal lobar degeneration, alcohol-related dementia and unspecified) | 9 long-term care facilities  June 2014 to May 2015 | PARO (seal robot) | 3 sessions (15 min)/week for 10 weeks | Outcomes: Engagement, mood state, agitation | Cohen Mansfield Agitation Inventory-Short Form (CMAI-SF) | Interactions with PARO were observed. The CMAI-SF was completed by facility care staff. | Participants with more severe agitation at baseline were also significantly more agitated at week 10 (*P<*.001). Results showed that less agitated patients showed greater behavioral engagement 10 (*P=*.009) and were less agitated (*P=*.045) at week 10. Low agitation and low cognitive impairment were both associated with better visual engagement (*P=*.006). Low cognitive impairment was also associated with more pleasure at the final week (*P=*.004). | Quantitative nonrandomized study 3.1. Yes (participants are representative of the target population, eligibility criteria are included) 3.2. Yes (measurements are appropriate to answer the research question) 3.3. Yes (outcome data are complete; 121 of 138 cases were included for one regression analysis and 123 of 138 cases were included for the four other regression models) 3.4. Yes (confounding factors are accounted for) 3.5. Yes (intervention was administered as intended) |
| Jøranson et al, 2015** [53] | Quantitative randomized controlled trial | N: 60 (40 women/ 20 men)  Age (years): 62-95 | Dementia or cognitive impairment | 10 Nursing homes  2012-2013 | PARO (seal robot) | 2 group sessions (30 min)/week for 12 weeks  Groups included 5-6 participants. | Outcomes: Agitation, depression | The Brief Agitation Rating Scale (BARS) (brief version of Cohen-Mansfield Agitation)   Cornell Scale for Symptoms of Depression in Dementia (CSDD) | For each participant, BARS and CSDD scores were measured at three points in time (baseline, postintervention, 3-month follow-up). | In the intervention group, agitation and depression decreased post-intervention and at 3-month follow-up. In the control group agitation and depression increased post-intervention and at 3-month follow-up. When comparing the change in the intervention group with control group from baseline to 3-month follow-up, the difference in effect estimates was significant for agitation (*P=*.048) and depression (*P=*.03). When comparing the change in the intervention group with control group from baseline to post-intervention, the difference in effect estimates was not significant for agitation (*P=*.10) and depression (*P=*.10). | Quantitative randomized controlled trial  2.1. Can't tell (the way randomization was performed is unclear) 2.2. Yes (groups were comparable at baseline) 2.3. Yes (outcome data is complete; data from 53 of 60 participants were included in analysis) 2.4. No (outcome assessors were not blinded to the intervention) 2.5. Yes (participants adhered to the assigned intervention) |
| Jøranson et al, 2016** [54] | Quantitative non  randomized study | N: 23 (16 women/ 7 men)  Age (years): 62-92 | Dementia | 5 Nursing homes  Study period unclear | PARO (seal robot) | 2 group sessions (30 min)/week for 12 weeks  Groups included 5-6 participants. | Outcome: Behavioral reactions of participants interacting with robots | No scales/scores used to measure outcome | Sessions of the second and tenth week were observed. An ethogram defining 18 different behaviors was developed to describe participants' behaviors. | Most frequent behavior registered was "Observing PARO", indicating interest from participants. Participants with mild to moderate dementia paid significantly more attention toward PARO (*P=*.02) compared to participants with severe dementia who tended to "[Observe] other things" (*P=*.04). However, patients with severe dementia observed PARO twice as much as they observed other participants, thus PARO was perceived as a meaningful occupation. From week 2 to week 10, “Smile/laughter toward other participants/[Activity leader]” increased significantly (*P=*.01), while “Conversation with PARO on the lap” significantly decreased (*P=*.01). | Quantitative nonrandomized study 3.1. Yes (participants are representative of the target population, eligibility criteria are included) 3.2. Yes (measurements are appropriate to answer the research question) 3.3. No (outcome data are not complete; 23 of 30 participants completed the study) 3.4. Yes (confounding factors are accounted for) 3.5. Yes (intervention was administered as intended) |
| K. Chen et al, 2020 [19] | Quantitative randomized controlled trial | N: 103 (82 women/ 21 men)  Age (years): 87.2 (mean) | Dementia | 7 Long-term care facilities  Study period unclear | Kabochan (humanoid robot) | Both groups went through 4 phases lasting 8 weeks each. The control group received the usual care through the entire 32 weeks. The intervention group received usual care for phases 1 and 3 and engaged with Kabochan during phases 2 and 4. Length of interaction with Kabochan unclear. | Primary outcomes: Neuropsychiatric symptoms, Caregiving distress, Depressive symptoms  Secondary outcomes : Cognitive ability, Functional dependency, Quality of life | Neuropsychiatric Inventory Questionnaire (NPI-Q) with symptom severity and caregiver distress subscales  Geriatric Depression Scale (GDS)  Hong Kong Montreal Cognitive Assessment 5-minute Protocol (MoCA)  Modified Barthel Index for Activities of Daily Living (ADL)  Quality of Life Alzheimer’s disease scale (QoL) | The NPI-Q and MoCA were used by therapists. The NPI-Q symptom severity subscale was completed by the main caregiver staff. The GDS and QoL scales were completed by a research assistant. The NPI-Q subscales, the GDS and QoL scale were used to measure outcomes at week 1, 8, 16, 24 and 32. ADL and MoCA were only used to measure outcomes at week 1 and 32. | There was a significant decrease in caregiver distress within the intervention group from week 8 (baseline) to week 16 (first intervention) (*P=*.01). Neuropsychiatric symptoms increased and became significantly more severe in the intervention group from week 16 to 24 (withdrawal) (*P=*.03). There were no significant changes in the severity of neuropsychiatric and depressive symptoms in the short (*P=*.33) and long term. (*P=*.68).  No statistically significant changes were observed for the 3 secondary outcomes from baseline to week 32 (cognitive ability (*P=*.07), functional dependency (*P=*.36), and quality of life (*P=*.78)). | Quantitative randomized controlled trial  2.1. Can’t tell (the way randomization was performed is unclear) 2.2. Yes (the groups were comparable at baseline) 2.3. Can't tell (outcome data might be incomplete, it is unclear whether all participants at baseline were included in the analysis) 2.4. No (outcome assessors were not blinded to the intervention) 2.5. Yes (participants adhered to the assigned intervention) |
| Kumazaki et al, 2018 [22] | Quantitative randomized controlled trial | N: 68 (23 girls/ 45 boys)  Age (years): 5-6 | Autism spectrum disorder (ASD) | Unclear. Recruitment from the Research Center for Child Mental Development, Kanazawa University  Study period unclear | CommU (humanoid robot) | Two groups were formed: a control group and an intervention group with both children with ASD and children with a typical development.  Intervention: Session 1: Interaction with human A  Session 2: Interaction with CommU or with human B  Session 3:  Interaction with human A | Outcome: Induction of joint attention (JA) by either a human or robotic agent (CommU) | No scales/scores used to measure outcome | Sessions were observed. Achievement of JA was defined as a response to the correct target within 3 seconds of initiation (by human or robot). Two trainers rated achievement of JA. Joint attention was rated a 1 (success) or a 0 (failure). Scores were added to obtain a total score for each interaction. | Exposure to the robot CommU increased JA among children with ASD (compared to JA during interaction with human). Children who previously interacted with the robot improved their performance in interacting with a human agent. The facilitative effect of joint attention due to CommU intervention was larger in children with autism than in typical developmental children.  (p-values unavailable) | Quantitative randomized controlled trial  2.1. Can't tell (the way randomization was performed is unclear) 2.2. Yes (the groups were comparable at baseline) 2.3. Yes (outcome data is complete, 66 of the 68 participants contributed to measures) 2.4. No (outcome assessors were not blinded to the intervention) 2.5. Yes (participants adhered to the assigned intervention) |
| L. Chen et al, 2020 [20] | Mixed method study | N: 3 (2 women/ 1 man)  Age (years): 78-86 | Dementia (Parkinson’s disease dementia, Alzheimer’s disease, Vascular dementia) | Day care  center for elderly.  July 15, 2018 -July 15, 2019. | Telenoid (humanoid robot) | 5 sessions (20 min) in total | Outcomes: uncanny valley hypothesis (the fact that a humanoid appearance might provoke the feeling of revulsion or dislike), social engagement and neuropsychiatric symptoms. | 12-items Neuropsychiatric inventory (NPI). | Scale/score were administered by researchers before and 48h after interaction. Interventions were observed. | No fear or dislike response to Telenoid´s human-like appearance and no change in neuropsychiatric symptoms were observed during interaction among the participants. Certain changes in social interaction and in mood were observed.   (p-values unavailable) | Mixed method study  5.1. No (the rationale for using both qualitative and quantitative data was not explained) 5.2. Yes (the different components of the study are effectively integrated to answer the research question) 5.3. Yes (outputs of the integration of quantitative and qualitative components are adequately interpreted) 5.4. Yes (divergence between qualitative and quantitative results are adequately addressed; no divergence is noted) 5.5. No (the different components of the study don’t adhere to the quality criteria of each tradition of the methods involved; the quality of the quantitative data is lacking) |
| Liang et al, 2017 [56] | Quantitative randomized controlled trial | N: 24 dyads of caregiver and care recipients with dementia (women/men ratio unavailable)  Age (years): 67-98 (care recipients with dementia) | Dementia | 2 dementia day care centers and participant's homes  Study period: unclear | PARO (seal robot) | The entire study lasted 12 weeks.  In the day care center: 2-3 sessions (30 min) for 6 weeks. The following 6 weeks, patients and their caregivers could use Paro at home whenever they preferred. | Primary outcomes: Cognition, agitation, neuropsychiatric symptoms and depressive symptoms.   Secondary outcomes: Behavioral, affective and social responses. | New Zealand version of the Addenbrooke's Cognitive Examination  Cohen-Mansfield Agitation Inventory Short-Form  Neuropsychiatric Inventory Brief Questionnaire Form  Cornell Scale for Depression in Dementia (CSDD) | Scales/scores were administered by researchers at 3-time-points (baseline, postintervention and follow-up).  Physical interactions, attention and communication with PARO were observed.  Blood pressure and salivary and hair cortisol levels were measured at 3 times points (baseline, postintervention and follow-up).  At week 6, caregivers were interviewed. | At the day care center, participants in the PARO group showed no statistically significant differences in agitated behavior (p>.05). Improvement in affective and social responses were observed. Participants in the PARO group were significantly happier (*P=*.04) and more likely to talk with the staff (*P=*.04) than participants in the control group. At home, PARO reduced anxiety, enhanced mood and encouraged positive interactions with visitors and family members, but improvement was not significant (p>.05). In both settings, patients who responded positively to PARO had significantly higher cognitive scores compared to those who showed neutral/ negative or mixed responses to PARO (*P=*.02). | Quantitative randomized controlled trial  2.1. Yes (randomization was appropriately performed; a computerized list was generated by a website) 2.2. Yes (the groups were comparable at baseline) 2.3. Yes (outcome data is complete; 24 of 30 dyads were included in analysis) 2.4. No (outcome assessors were not blinded to the intervention) 2.5. Yes (participants adhered to the assigned intervention) |
| Moyle et al, 2016 [8] | Mixed method study | N: 5 women  Age (years): 68-98 | Dementia | 1 Nursing home  Study period: unclear | CuDDler (teddy bear robot) | 3 sessions (30 min)/week for 5 weeks | Outcomes: Engagement, agitation and emotional response. | Cohen-Mansfield Agitation Inventory Short-Form (CMAI-SF)  Observed Emotions Rating Scale (OERS) | Scales/scores were administered by staff before and after the interactions with CuDDler.  Individual semi-structured interviews (15 min) were administered by the intervention facilitator at the end of the study. | Overall, CuDDler had different effects on the five participants, but it provided the opportunity for the participants to engage in social interaction and to reduce agitation (reported in 3 patients). Positive emotional responses (laughs/smiles, pleasure) were observed in all participants.   (p-values unavailable) | Mixed method study 5.1. No (the rationale for using both qualitative and quantitative data is not explained) 5.2. Yes (the different components of the study are effectively integrated to answer the research question) 5.3. Yes (outputs of the integration of quantitative and qualitative components are adequately interpreted) 5.4. Yes (divergence between qualitative and quantitative results are adequately addressed) 5.5. Yes (the different components of the study adhere to the quality criteria of each tradition of the methods involved) |
| Moyle et al, 2017 [57] | Quantitative randomized controlled trial | N: 415 (314 women/101 men)  Age (years): ≥ 60 | Dementia | 28 Long-term care facilities  June 2014 to May 2015 | PARO (seal robot) | 3 sessions (15 min)/week for 10 weeks | Primary outcomes: Agitation, engagement and mood states after 10-week intervention  Secondary outcomes: Agitation, engagement and mood states at weeks 1 and 5 of intervention and postinterven-tion | Cohen-Mansfield Agitation Inventory Short-Form | Participants were observed to evaluate outcomes. Scale/score were administered by facility care staff.  Assessments were made at 5-time points (baseline, weeks 1, 5, 10, 15). | Primary outcomes: When comparing the use of PARO vs. usual care, no statistically significant differences in agitation after 10-week intervention were noted with CMAI-SF score (*P=*.34). However, a significant improvement in agitation was observed on video recording (*P=*.008). Compared with the plush toy, participants using Paro showed engagement (verbally: *P=*.01; visually: *P<*.0001). Decrease in neutral affect were significant in both PARO group (*P=*.02) and the plush toy group (*P=*.002) compared to usual care. Pleasure was significantly better in the PARO group compared to usual care (*P=*.008).  Secondary outcomes: Positive effects (agitation, engagement and mood states) at weeks 1 and 5, but little sustainability beyond the intervention period. The effects of PARO are limited to the intervention period. | Quantitative randomized controlled trial  2.1. Yes (randomization was appropriately performed; a computer-generated sequence was used) 2.2. Yes (the groups were comparable at baseline) 2.3. Yes (outcome data is complete; data from all participants were included in analysis) 2.4. Yes (outcome assessors were blinded to the intervention) 2.5. Yes (participants adhered to the assigned intervention)" |
| Naganuma et al, 2015 [58] | Qualitative study | N: Unclear (at least 2 women)  Age (years): unclear | Dementia | Nursing home  Study period: unclear | AIBO (dog robot) | A single session with duration unclear. | Outcomes: Interaction between residents | No scales/scores used to measure outcome | Observation of participants by researchers. | AIBO effectively produced a cue to initiate communication between residents in the nursing home. | Qualitative study - Qualitative description 1.1. Yes (the qualitative approach is appropriate to answer the research question) 1.2. Can't tell (data collection methods are not described) 1.3. Can't tell (findings might not be adequately derived from data, no analysis method is mentioned) 1.4. No (the interpretation of results cannot be supported by data) 1.5. No (links between qualitative data sources, collection, analysis and interpretation are unclear) |
| Narita et al, 2016 [59] | Quantitative non  randomized study | N: 3 (2 women/ 1 man)  Age (years): 27-54 | Schizophrenia | Day care program  Study period: unclear | AIBO (dog robot) | 1 session (60 min)/week for 8 weeks | Outcomes: Anxiety, positive and negative symptoms | State-Trait Anxiety Inventory (STAI)  Positive and Negative Syndrome Scale (PANSS). | Scales/scores and interviews administered by psychiatrists pre and post intervention. | Overall, improvements in anxiety, emotional withdrawal, social avoidance, motor retardation and depression.  (p-values unavailable) | Quantitative nonrandomized study 3.1. No (participants are not necessarily representative of the target population, the reasons of inclusion are not clear) 3.2. Yes (measurements are appropriate to assess outcome) 3.3. Yes (outcome data are complete) 3.4. Yes (confounding factors are accounted for and possible bias is discussed) 3.5. Yes (intervention was administered as intended) |
| Petersen et al, 2017 [60] | Quantitative randomized controlled trial | N: 61 (47 women/ 14 men)  Age (years): ≥ 65 (average: 83.4) | Dementia | 5 urban secure dementia units  Study period: unclear | PARO (seal robot) | 3 sessions (20 min)/week for 12 weeks. | Outcomes: dementia -related symptoms (anxiety and depression) | Rating for Anxiety in Dementia (RAID)  Cornell Scale for Depression in Dementia (CSDD)  Global Deterioration Scale (GDS) | Objective data (pulse oximetry, pulse rate and galvanic skin response) were collected before and after each exposure to the robotic pet by staff and trained facility nurses.  Scales/scores were administered by trained facility nurses pre and post intervention. | Changes in anxiety (RAID) (*P=*.003) and depression levels (CSDD) (*P=*.001) were significantly greater in the intervention group compared with control, while changes in global deterioration (GDS) (*P=*.95) were not.  Changes in pulse oximetry (*P=*.0001), pulse rate (*P=*.0001) and galvanic skin response (*P=*.0005) were also significantly greater in the intervention group. | Quantitative randomized controlled trial  2.1. Yes (randomization was appropriately performed, groups were assigned by the toss of a coin) 2.2. Yes (the groups were comparable at baseline) 2.3. Can't tell (outcome data might be incomplete, it is unclear whether all participants at baseline were included in the analysis) 2.4. No (outcome assessors were not blinded to the intervention) 2.5. Yes (participants adhered to the assigned intervention) |
| Pu et al, 2020 [61] | Qualitative study | N: 11 (9 women/ 2 men)  Age (years): ≥ 65 | Dementia (Alzheimer's disease, vascular dementia and unspecified dementia) and chronic pain. | 3 Residential care facilities  January 2018 - January 2019 | PARO (seal robot) | 5 sessions (30 min)/week for 6 weeks | Outcomes: Perceptions, therapeutic effects and limitations of PARO | No scales/scores used to measure outcome | Individual semi-structured interviews administered by researchers at the end of the study. | Participants reported positive attitudes toward PARO: improvement of mood, comfort and distraction from their pain. However, some of them reported limitations concerning PARO's functions and programming. | Qualitative study - Qualitative description 1.1. Yes (the qualitative approach is appropriate to answer the research question) 1.2. Yes (the qualitative data collection methods are adequate to address the research question) 1.3. Yes (the findings are adequately derived from the data, a six-step inductive thematic analysis was followed) 1.4. Yes (the interpretation is sufficiently substantiated by data) 1.5. Yes (links between qualitative data sources, collection, analysis and interpretation are coherent) |
| Rakhymbayeva et al, 2021 [62] | Mixed method study | N: 11 (1 girl/ 10 boys) Age (years) : 4-11 | Autism spectrum disorder (ASD) and attention deficit hyperactivity disorder. | Rehabilitation center  Study period: unclear | NAO (humanoid robot) | At least 7 sessions (15 min) on separate days. There was a variation in the number of sessions between participants. | Outcomes: social engagement, valence (emotional responses) and eye gaze duration. | 5-point Likert scales developed to assess engagement and valence. | Observation of participants by researchers.  Scales/ scores administered by researchers in all sessions.  Semi-structured interviews were administered by researchers with parents or caregivers after sessions. | Children engaged over multiple sessions, notably in familiar situations and when the robot was used in activities preferred by the child. No significant difference in engagement duration between the first and the last session was found. Valence scores and eye gaze time did not reveal any significant differences between sessions.   (p-values unavailable) | Mixed method study  5.1. No (the rationale for using both qualitative and quantitative data is not explained) 5.2. Yes (the different components of the study are effectively integrated to answer the research question) 5.3. Yes (outputs of the integration if quantitative and qualitative components are adequately interpreted) 5.4. Yes (no divergence between qualitative and quantitative results were found) 5.5. Yes (the different components of the study adhere to the quality criteria of each tradition of the methods involved) |
| S. Chen et al, 2020 [55] | Mixed method study | N: 20 (13 women/ 7 men)  Age (years): ≥ 65 | Depression | 4 Long-term care facilities   Study period: unclear | PARO (seal robot) | Participants kept Paro full-time for 8 weeks. | Outcomes: Depression, loneliness, quality of life | Geriatric Depression Scale (GDS-SF)  UCLA Loneliness Scale Version 3 (UCLA-3)  World Health Organization Quality of Life Questionnaire for older adults (WHO-QOL-OLD). | Scales/ scores administered by researchers at 4 time points (a week before the start of the 8-week observation (T1), at the end of the 8-week observation (T2), at the mid-point of the PARO intervention (T3) and at the end of the 8-week Paro intervention (T4).  Semi-structured interviews were administered by researchers (30-40 min) after the PARO intervention. | Statistically significant reduction in depression (T2 vs. T3: *P<*.001; T2 vs. T4: *P<*.001; T3 vs. T4: *P=*.02), loneliness (T2 vs. T3: *P<*.001; T2 vs. T4: *P<*.001; T3 vs. T4: *P=*.02) and an improvement in quality of life (T2 vs. T3: *P=*.001; T2 vs. T4: *P<*.001; T3 vs. T4: *P=*.33) between the observation and the intervention stages. | Mixed method study  5.1. No (the rationale for using both qualitative and quantitative data is not explained) 5.2. Yes (the different components of the study are effectively integrated to answer the research question) 5.3. Yes (outputs of the integration if quantitative and qualitative components are adequately interpreted) 5.4. Yes (no divergence between qualitative and quantitative results were found) 5.5. Yes (the different components of the study adhere to the quality criteria of each tradition of the methods involved) |
| Sato et al, 2020 [63] | Qualitative study | N: 9 (proportion among women and men unclear)  Age (years): unclear | Dementia and/or schizophrenia. | Psychiatric hospitals and day services in geriatric health facilities  Study period: unclear | Pepper (humanoid robot) | The number of sessions and their duration was unclear. Interactions with Pepper throughout the rehabilitation and recreation sessions in long-term care settings. | Outcomes: Reaction/ attitudes toward Pepper; interaction and health benefits | No scales/scores used to measure outcome | Observation of participants by researchers. | Both inaction (ie,no change in behavior) and coaction (ie, reaction with visible responses) occurred during the interaction between Pepper and patients. The interactions elicited good connections, but some difficulties were noted too (eg, patients were sleepy during the intervention, patients were unable to hear). Pepper was successful to keep older patients active and healthy through health promotion activities. | Qualitative study - Qualitative description 1.1. Yes (the qualitative approach is appropriate to answer the research question) 1.2. Yes (the qualitative data collection methods are adequate to address the research question; observation, notes, field tests, recorded videos were analyzed) 1.3. Yes (the findings are adequately derived from the data, analysis was case-oriented) 1.4. Yes (the interpretation is sufficiently substantiated by data) 1.5. Yes (links between qualitative data sources, collection, analysis and interpretation are coherent) |
| Valentí Soler et al, 2015 [64] | Quantitative randomized controlled trial | N:121 (99 women/22 men) in phase 1 and N:127 (109 women/18 men) in phase 2  Age: 58-100 | Dementia (Alzheimer's disease, mixed dementia, Parkinson's disease dementia, dementia with Lewy bodies, Frontotemporal dementia). | Nursing home and day care center  Study period: unclear | PARO (seal robot), NAO (humanoid robot). | 2 sessions (30-40 min)/week for 12 weeks  There were two phases of 3 months each with a washout phase in between. | Outcomes: Behavior changes, apathy and quality of life | Global Deterioration Scale (GDS)  Neuropsychiatric Inventory (NPI)  Apathy Scale for Institutionalized Patients with Dementia Nursing Home version (APADEM-NH)  Apathy Inventory (AI)  Quality of Life Scale (QUALID) | Scores/scales were administered by blinded raters at baseline and follow-up.  Nursing staff was interviewed at baseline and follow-up. | Phase 1: In the nursing home, all groups showed a statistically significant increase in GDS scores. Both robot groups showed significant reduction in apathy (PARO; NAO). Participants in the NAO group were more delusional and participants in the PARO group showed signs irritability/lability, hallucinations and disinhibition, but a reduction in night-time behavior disturbances. In the day care center, NPI irritability and total score improved.  Phase 2:  The PARO group slightly worsened in quality of life.  (p-values unavailable) | Quantitative randomized controlled trial  2.1. Yes (randomization was appropriately performed, a six-sided die was used) 2.2. Yes (the groups were comparable at baseline) 2.3. Yes (outcome data is complete, overall measures were obtained from most participants present at baseline) 2.4. Yes (outcome assessors were blinded to the intervention) 2.5. Yes (participants adhered to the assigned intervention) |
| Wagemaker et al, 2017 [65] | Quantitative non  randomized study | N: 5 (3 women/ 2 men)  Age (years): 59-70 | Intellectual disabilities | Residential mental healthcare institution  Study period: unclear | PARO (seal robot) | Participants were able to use the robot PARO whenever they liked for 4 weeks, and the same ritual was applied for interactions with the plush toy. | Outcomes: Mood and alertness | Visual mood scale (3 smileys: sad/angry, neutral or happy)  Alertness Observation List (AOL) | Scores/scales were administered twice a day. Mood was self-reported and rated by supervisors. Alertness (AOL) was assessed by daily supervisors.  Informal observations were made by daily supervisors. | Of 5 participants, only one showed statistically significant improvements in mood (self-reported: *P=*.08; rated by supervisors: *P<*.05) and alertness at the end of the treatment (*P<*.01). | Quantitative nonrandomized study 3.1. Can't tell (participants might not be representative of the target population, eligibility criteria are not clear) 3.2. Yes (measurements are appropriate to assess outcome) 3.3. Yes (outcome data are complete; an average of 94.4 (out of a possible 112) measurements per participant was obtained) 3.4. Yes (confounding factors are accounted for) 3.5. Yes (intervention was administered as intended) |

† P-values were rounded-up in accordance with the instructions for authors

†† MMAT allows quality assessment of quantitative descriptive studies, qualitative and mixed methods studies. Agreement was defined when both raters gave a positive (yes) or a negative (cannot tell, no) score. Full version of the MMAT is available from: <http://mixedmethodsappraisaltoolpublic.pbworks.com/w/file/fetch/146002140/MMAT_2018_criteria-manual_2018-08-08c.pdf> . Reference: Hong QN, Pluye P, Fàbregues S, Bartlett G, Boardman F, Cargo M, Dagenais P, Gagnon M-P, Griffiths F, Nicolau B, O’Cathain A, Rousseau M-C, Vedel I. Mixed Methods Appraisal Tool (MMAT), version 2018. Registration of Copyright (#1148552), Canadian Intellectual Property Office, Industry Canada.

* Articles by Barrett et al., Casey et al. and D’Onofrio et al. discuss different outcomes of the same study.

**Articles by Jøranson et al. from 2015 and 2016 discuss different outcomes of the same study.

(Complete references of the included papers are available in the full-text article.)
